# Diagnostic value of multi-modal ultrasound combined with intracavernosal injection testing for concurrent erectile dysfunction and penile curvature

**DOI:** 10.1093/sexmed/qfag002

**Published:** 2026-01-30

**Authors:** Muyi Mao, Jiahui Peng, Lujing Li, Senbao Tan, Huitong Lin, Zuofeng Xu

**Affiliations:** Department of Medical Ultrasonics, The Seventh Affiliated Hospital of Sun Yat-sen University, Zhenyuan Road, Guangming District, Shenzhen 518000, Guangdong, China; Department of Medical Ultrasonics, The Seventh Affiliated Hospital of Sun Yat-sen University, Zhenyuan Road, Guangming District, Shenzhen 518000, Guangdong, China; Department of Medical Ultrasonics, The Seventh Affiliated Hospital of Sun Yat-sen University, Zhenyuan Road, Guangming District, Shenzhen 518000, Guangdong, China; Department of Andrology, The Seventh Affiliated Hospital of Sun Yat-sen University, Zhenyuan Road, Guangming District, Shenzhen 518000, Guangdong, China; Department of Medical Ultrasonics, The Seventh Affiliated Hospital of Sun Yat-sen University, Zhenyuan Road, Guangming District, Shenzhen 518000, Guangdong, China; Department of Medical Ultrasonics, The Seventh Affiliated Hospital of Sun Yat-sen University, Zhenyuan Road, Guangming District, Shenzhen 518000, Guangdong, China

**Keywords:** multi-modal ultrasound, intracavernous injection, erectile dysfunction, penis curvature

## Abstract

**Background:**

The combination of Intracavernosal Injection (ICI) and Color Doppler Duplex Ultrasonography (CDDU) has emerged as a novel approach for evaluating erectile dysfunction (ED) in recent years.

**Aim:**

To evaluate the diagnostic value of multi-modal ultrasound integrated with ICI in patients with ED concomitant with penile curvature.

**Methods:**

Eighty-two ED patients were prospectively enrolled in this observational study between April 2021 and February 2025. Multimodal ultrasound, including high frequency ultrasound, CDDU, and shear wave elastography were used to evaluate the penile hemodynamic and structural parameters pre- and post-ICI of Papaverine Hydrochloride. The peak systolic velocity (PSV), end diastolic velocity (EDV), and resistance index (RI) of the corpus cavernosum artery and SWE-derived Young’s modulus values (YM) of the corpus cavernosum were examined. Participants were stratified by curvature presence, with subgroup analyses by different angle (<15°, 15-30°, 31-60°, >60°) and different direction (ventral/lateral, left/right).

**Outcomes:**

The differences of penile hemodynamic and structural parameters pre- and post-ICI of all ED patients, and different subgroups of patients with penile curvature were evaluated.

**Results:**

(1) Eighty-two men with a median age of 34 (21-67) years were evaluated, including 34 patients (41.46%) diagnosed as ED with penile curvature and 48 patients (58.54%) diagnosed as ED without penile curvature. (2) Using High-frequency ultrasound, Peyronie’s disease was detected in 8 patients (9.76%). (3) Following ICI, compared to pre-ICI measurements, both groups exhibited significant increases in PSV (*P* < .001) and EDV (*P* = .012) of cavernous artery and a reduction in YM (*P* < .001). (4) Patients in moderate/severe curvature group showed higher EDV (*P* = .01) and lower RI (*P* = .02) than no/mild curvature groups, with Significant differences. (5) Significant differences in pre-ICI YM were observed among patients categorized by penile curvature direction (*P* = .03).

**Clinical Implications:**

We provide a non-invasive, reproducible method for the integrated assessment of ED complicated with penile curvature by multi-modal ultrasound combined with ICI, and correlate pre-intervention biomechanical and vascular profiles with post-erection anatomical outcomes in ED patients.

**Strengths & Limitations:**

The strength of the study lies in its unique approach to evaluate patients with ED concomitant with penile curvature, making the clinical evaluation more complete and precise. However, the limited sample size restricts generalizability and further research.

**Conclusion:**

Multi-modal ultrasound combined with ICI provides a non-invasive, reproducible method for the integrated assessment of ED complicated with penile curvature.

## Introduction

Erectile dysfunction (ED)，clinically defined as the persistent inability to achieve or sustain penile erection adequate for satisfactory sexual activity over 6 consecutive months, represents the most common form of male sexual dysfunction.^1^ ED demonstrates a significant global prevalence, emerging as a substantial public health challenge across most nations. Particularly in Asian populations, epidemiological studies report prevalence rates ranging from 27%-68%.^2^ Clinically, ED can be divided into three primary etiological categories: organic, psychogenic, and mixed subtypes. The pathophysiological mechanisms of organic ED include neurogenic disorders, endocrine abnormalities, vasculogenic impairments, and medication-induced complications, and so on.^3^ ED not only damages the physical and mental health of patients, but also affects their family stability and living quality.

In recent years, the diagnosis and treatment of male ED has gradually formed a clinical consensus.^4–6^ Traditional diagnostic methods for ED include medical history collection, physical examination, laboratory testing, etc. During history taking, international Index of Erectile Function (IIEF), and Erection Hardness Score (EHS) are mandatory for objective erectile function quantification. Laboratory tests include fasting blood glucose, glycosylated hemoglobin, fasting blood lipids, and serum testosterone levels. Emerging diagnostic modalities supplement conventional methods, such as penile erection hardness testing, penile cavernosography, intracavernosal injection (ICI) of vasoactive agents, and combination assessment with penile color duplex Doppler ultrasonography (CDDU). The combined ICI-CDDU protocol has gained widespread adoption for vascular ED assessment and is considered important for penile hemodynamics evaluation.^7^ In recent years, shear wave elastography (SWE) has progressively emerged as a novel diagnostic technique in penile disease management and ED evaluation.^8–10^

Penile curvature coexistin with ED represents a distinct clinical type of ED with unique pathophysiological features. Penile curvature is broadly classified into congenital and acquired types. Congenital cases frequently correlate with urethral malformations, with an incidence rate of 25%,^11^ while acquired penile curvature cases are mostly caused by trauma, infections, fibrotic scarring, or Peyronie’s disease.^12^^,^^13^ Notably, acquired penile curvature often present organic structural abnormalities which may directly contribute to ED pathogenesis. Curvature severity classification based on curvature angles remains heterogeneous across studies,^14–17^ including three-tier system (mild: <30°, moderate: 30°-60°, severe:>60°), four-tier system (insignificant: <20°, mild:20°-30°, moderate:30°-60°, severe: >60°), or alternative three-tier system (slight:<15°, moderate:15°-30°, severe: >30°). Despite no consensus has been achieved, clinical observations consistently indicate that patients with penile curvature angle <30° has little impact on sexual intercourse, while when penile curvature angle >30°, penetrative capacity will be affected.^18^ Therefore, in patients presenting with ED and concurrent penile curvature, especially for patients with a large curvature angle >30°, clinical attention should be paid not only to erectile function restoration but also to the penile curvature anatomical correction.

As previously mentioned, penile curvature coexisting with ED represents a distinct clinical subtype of ED. To enable a comprehensive assessment, can we integrate the current gold standard diagnostic method—the combined ICI-CDDU protocol for evaluating this complex condition? Beyond its established role in ED assessment, does the ICI-CDDU approach offer additional diagnostic value for characterizing penile curvature? Therefore, our study aimed to explore the role of ICI combined with multi-modality ultrasound in dignosing ED with penile curvature and to establish an evidence-based foundation for therapeutic decision-making in this patient population.

## Material and methods

### Participants

This prospective study was approved by the Institutional Review Board, written informed consent was obtained from all participants prior to enrollment. The study cohort comprised 82 consecutive male patients recruited from the department of andrology between April 2021 and February 2025.

Inclusion criteria:

1. Adult males (age > 18 years) with marital status or long-term stable sexual partnership.
2. Primary clinical presentations of persistent erectile hardness reduction, erectile maintenance difficulty, and coital failure (≥3 months duration).
3. Objective penile curvature during erection with varying degrees.
4. Normal secondary sexual characteristics development and no obvious abnormalities in the external genitalia.
5. Relevant biochemical parameters within normal ranges: including serum testosterone, follicle stimulating hormone, luteinizing hormone, estradiol, prolactin, blood glucose, blood lipids, hepatic, and renal function tests, as well as blood and urine routine tests.
6. The IIEF-5 score was less than 22 points.
7. No contraindications for high-frequency ultrasound, color Doppler ultrasound, and shear wave elastography examintions.
8. Signed informed consent documentation.

Exclusion criteria:

1. Acute cardiovascular and cerebrovascular events within 6 months, such as stroke or myocardial infarction.
2. Advanced hepatic/renal function insufficiency (Child-Pugh C, eGFR <30 mL/min/1.73m^2^).
3. Congenital or acquired genital abnormalities.
4. Uncontrolled hypertension (BP >160/100 mmHg despite therapy).
5. Recent nitrate or similar medications therapy.
6. Post-surgical or traumatic ED (eg, post-prostatectomy).
7. Severe mental illnesses or psychological abnormalities (eg, untreated depression, anxiety, schizophrenia)
8. Substance abuse history (eg, illicit drug or alcohol dependence).
9. ED-specific treatments within 3 months.
10. Ultrasound contraindications or impaired cooperation.
11. ICI therapy contraindications.

### Instruments and technique

All studies were performed by experienced andrologists and radiologists (andrologists with > 5 years of clinical experiences and radiologists with > 10 years of specialized experiences). The andrologists were primarily responsible for andrological evaluations and ICI procedures, while the radiologist conducted multi-modal ultrasound examinations.

A Canon i900 color Doppler ultrasound diagnostic instrument (Canon i900, Canon Medical Systems Corporation, Tochigi Prefecture, Japan, 2021) with a high-frequency linear array probe(L18-3, 4.5-9.0 MHz) was used, Shear wave elastography was employed to quantify penile stiffness.

All procedures were performed in an isolated, quiet and warm room，participants layed in a supine position with penile dorsum gently opposed to the abdominal wall.

#### Intracavernous injection protocal

A dose of 30-50 mg Papaverine was administered subcutaneously through the corpus cavernosum of the penis using standard aseptic technique in order to induce pharmaceutical erection. Specific Procedure: After routine skin disinfection, the medication is injected into one of the corpora cavernosa. Pressure is applied for several minutes to prevent hematoma formation, followed by the application of audiovisual sexual stimulation to facilitate penile erection. The injection site is selected at the midportion of either lateral side of the penis to avoid the dorsal neurovascular structures, urethra, and glans penis. A 30-gauge (30G) needle is generally recommended to minimize trauma to the corpus cavernosum, with careful attention to the depth of insertion to avoid injection into the urethra or subcutaneous tissue. Supplemental Dosing Protocol: The baseline dose for each patient is 30 mg. If, after 10 minutes of administration, our assessment indicates that the patient still has no significant erection, a supplemental dose of 10-20 mg will be considered. This process is repeated until the total cumulative dose reaches 50 mg.

#### Method of multi-modal ultrasound examination

The linear array transducer (7.5-12 MHz) was placed with minimal compression on the penile ventral side to scan the corpora cavernosa of the penis (CCP).

High-frequency ultrasonography: The hierarchical structure of the penile corpora cavernosa was clearly displayed through high-frequency ultrasound, enabling concurrent detection of Peyronie’s diseases, corporal fibrosis, calcification, and penile lesions, etc.

**Figure 1:**
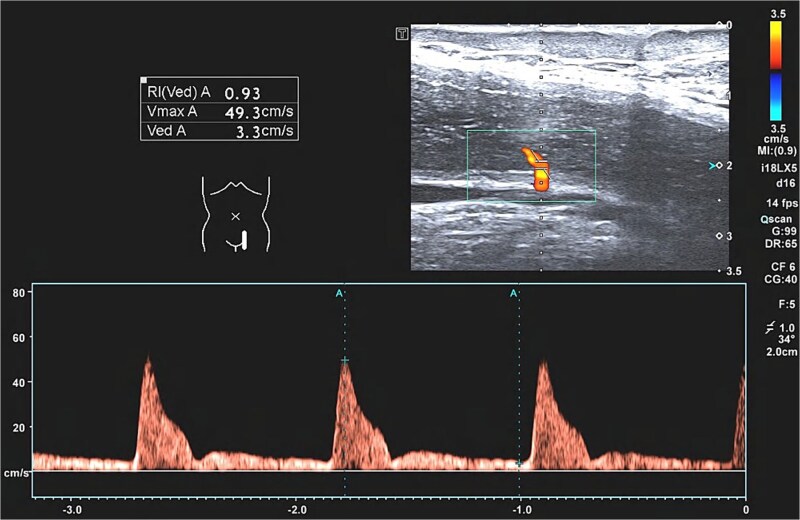
Color doppler ultrasound examination and measurement of CCP root penile artery.

Color Doppler ultrasound examination: Through Doppler ultrasound observation and measurement of blood flow spectrum of bilateral penile root corpus cavernosum arteries in both flaccid state and pharmacologically induced erectile state following ICI，hemodynamic parameters of bilateral CCP root penile artery, including peak systolic velocity (PSV), end diastolic velocity (EDV), and resistance index (RI) were systematically measured and recorded (Figure 1).

**Figure 2:**
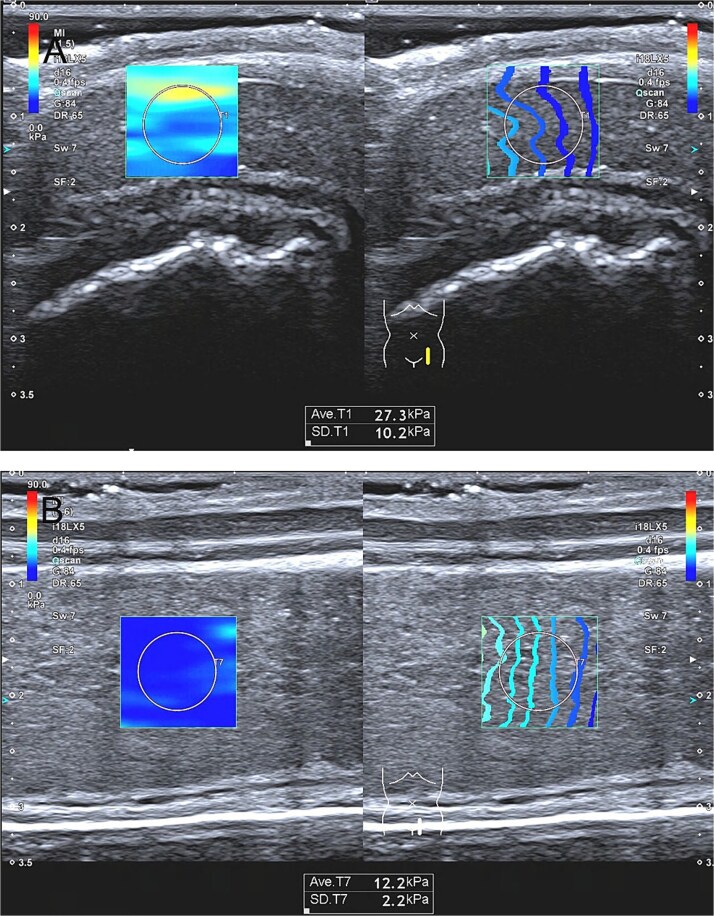
Shear wave elastography and quantitative elasticity measurements of penile corpus cavernosum. (A) Quantitative elasticity measurements of penile corpus cavernosum in the flaccid states. (B) Quantitative elasticity measurements of penile corpus cavernosum in ICI-induced erectile states.

Shear wave elastography and Young’s Modulus Acquisition: Quantitative elasticity measurements of bilateral penile corpus cavernosum were performed in both flaccid and ICI-induced erectile states. The ultrasound probe was positioned gently on the ventral surface of the penis with a longitudinal scanning plane, avoiding penile corpus cavernosum artery, then activating SWE mode. Following stabilization of the elastic maps (≥90% color fill), a standardized region of interest was positioned approximately 1 cm subjacent to the skin surface, the Young’s modulus (YM) of this region was acquired after image freezing. Multiple measurements (at least 5 replicates, each replicate lasting for 5-8 seconds) were acquired at defined intervals pre- and post-ICI administration (5, 10, 15, 20, and 30 minutes), with protocol adaptation for patients exhibiting shorter erection durations. Final elasticity values were calculated as the mean of all valid measurements, expressed in kilopascals (kPa) (Figure 2).

### International index of erectile Function-5 scoring criteria

(1) Normal erectile function: 22-25 points;
(2) Mild ED: 12-21 points;
(3) Mild ED: 8-11 points;
(4) Severe ED: ≤7 points.

### Penile rigidity grading

Penile rigidity was assessed using the Schramek penile hardness classification system, which defineed 5 grades: Schramek Grade V (full erection with maximal rigidity) Grade IV (suboptimal rigidity), Grade III (partial rigidity), Grade II (minimal rigidity), and Grade I (no response). Correlation with EHS is as follows: EHS grade 4 (full rigidity sustained ≥10 minutes) t corresponds to Schramek grade V, When EHS grades 3, 2, and 1 (sustained ≥10 minutes) align with Schramek grades IV, III, and II，respectively.

### Penile curvature assessment protocal

The subjects were placed in the supine position. Following pharmacologically-induced penile erection by ICI of papaverine, the direction of penile curvature was observed, and the angle of curvature was measured and recorded using a standard goniometer. All measurements were conducted in the presence of two individuals, with both parties cross-verifying each other’s results.

The direction of penile curvature in the erect state was documented as follows: leftward curvature, left-dorsal curvature, left-ventral curvature, rightward curvature, right-dorsal curvature, ventral curvature.

**Figure 3:**
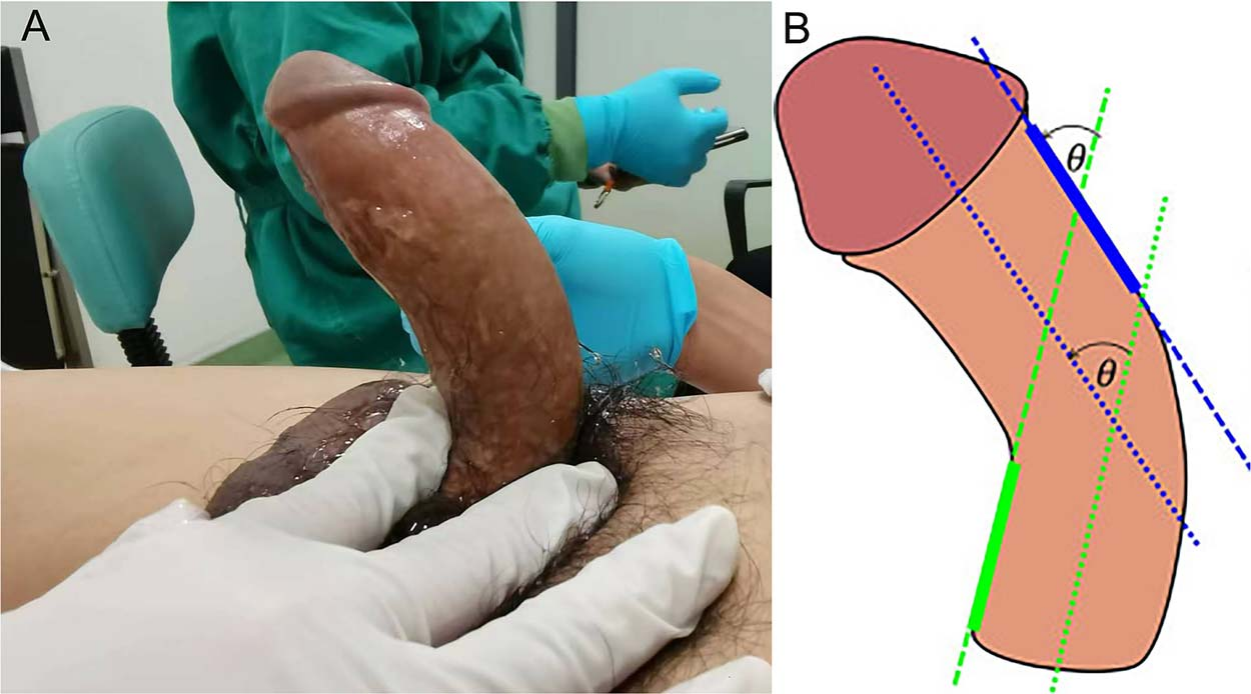
The measurement of angulation of penile curvature in the erect state. Angle θ was the horizontal plane angulation of penile curvature. The sagittal plane angulation was acquired by the similar methods.

The angulation of penile curvature in the erect state was measured using the penoscrotal junction as the anatomical baseline; the curvature angle was geometrically defined as the deviation between the penis glans apex and the anatomical baseline reference, quantified in two orthogonal planes: horizontal plane angulation and sagittal plane angulation, with measurements recorded through standardized goniometric protocols (Figure 3).

Based on expert recommendations and practical conditions, patients were divided into four groups according to of penile curvature angle: insignificant curvature group (<15°), mild curvature group (15°-30°), moderate curvature group (31°-60°), severe curvature group (>60°). The cohort comprised 50 cases (60.98%) in insignificant curvature group, 12 cases (14.63%) in mild curvature group, and 20 cases (24.39%) in the combined moderate or severe group.

### Statistical analyses

Statistical analyses were performed using SPSS software (version 26.0, BM Corporation, USA, 2018).

Continuous variables underwent normality testing via the Kolmogorov–Smirnov test. Descriptive statistics for continuous variables were presented as mean ± standard deviation (SD),or median (inter-quartile range). Intergroup comparisons of continuous variables were performed using Student’s *t*-test for normally distributed data and Mann–Whitney U test for non-parametric distributions. Paired *t*-tests or Wilcoxon signed-rank tests were conducted for pre-post comparison of cavernosal blood flow and elasticity parameters following ICI. Independent samples *t*-tests or Mann–Whitney U tests were used to compare cavernosal hemodynamic and elasticity parameters between curvature group and non-curvature groups. Subgroup stratification based on penile curvature angulation and orientation enabled comparative assessment of vascular parameters, and elasticity metrics using Kruskal–Wallis test. ROC curve analysis was employed to assess vascular and elastic parameters in the significant penile curvature group, while the chi-square test was used to explore the relationship between the etiology of ED and penile curvature. Statistical significance was defined as *P* < .05 for all analyses.

## Result

### Participant and characteristics

This study enrolled 82 patients ranging in age from 21 to 67 years (median age of 34 years). All patients fulfilled established diagnostic criteria for ED. After ICI of papaverine, 63 patients (76.83%) demonstrated penile rigidity grade ≥ Schramek IV; 19 patients (23.17%) exhibited grade below IV, Erection duration analysis revealed 57 patients (69.51%) maintained erections >15 minutes and 25 patients (30.49%) with erecting duration <15 minutes. The cohort comprised 34 patients (41.46%) diagnosed with concomitant ED and penile curvature, and 48 patients (58.54%) presented with ED without penile curvature.

### Multi-modal ultrasound evaluation of penile architecture and hemodynamics

#### High-frequency ultrasonography

High-frequency ultrasonography effectively visualizes penile anatomical stratification, delineating from superficial to deep layers: skin layer, fascial layer, tunica albuginea, and corpora cavernosa structure**.** Among 82 patients, 8 patients (9.76%) with Peyronie’s disease were detected by high-frequency ultrasonography. The mean maximum diameter of penile plaques was approximately 21.22 ± 7.65 mm. Notably, 6 of these 8 PD patients exhibited varying degrees of erectile asymmetry during pharmacologically-induced tumescence with lesions localized to ipsilateral to penile curvature in 5 cases and contralateral in 1 case.

#### Color duplex Doppler ultrasonography

All 82 patients underwent comprehensive color Doppler ultrasound examination and bilateral penile root artery hemodynamic parameters were systematically recorded. The average values of hemodynamic parameters were derived from triplicate measurements to ensure reliability. During full erection, the median values demonstrated: PSV = 52.83 cm/s (16.20 cm/s-108.05cm/s), EDV = 0.44 cm/s (0.00-3.06 cm/s), and RI of penile root artery = 0.99(0.90-1.00).

#### Shear wave elastography

A subset of 34 patients underwent systematic shear wave elastographic scans and assessments, pre-/post-pharmacologically-induced erection. Bilateral corpora cavernosa stiffness was quantitatively evaluated, with median YM value of 22.88kpa (7.19kpa-49.75kpa) in the flaccid state and 7.07 kpa (3.42kpa-39.89kpa) in the tumescent state.

### Hemodynamic and biomechanical alterations of penile post-papaverine ICI

**Figure 4:**
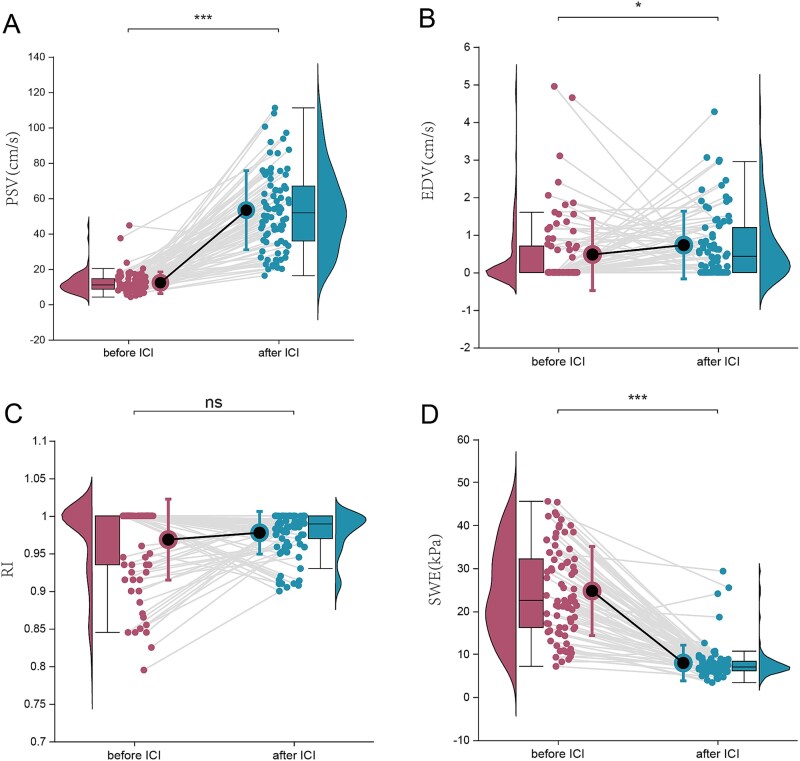
The hemodynamic and biomechanical alterations of penile post-papaverine ICI. Pre-/post-injection comparisons demonstrated that significant elevation in PSV (A) of cavernous artery with marginal EDV increase (B), while RI showed non-significant variation (C). Concurrently, corporal biomechanical assessment revealed that the penile YM values decreased following vasoactive injection (D).

Pre-/post-injection comparisons demonstrated that significant elevation in PSV of cavernous artery with marginal EDV increase (*P* < .05), while RI showed non-significant variation (*P* > .05). Concurrently, corporal biomechanical assessment revealed that the penile YM values decreased following vasoactive injection (*P* < .05) (Figure 4). Detailed comparative metrics are presented in Table 1.

**Table 1:** The blood flow parameters and elasticity values pre- and post-ICI.

	No.	PSV (cm/s)	EDV (cm/s)	RI	YM (kpa)
Pre-ICI	82	10.95 (8.48, 14.58)	0.00 (0.00，0.70)	1.00 (0.94，1.00)	22.88 (16.24, 33.55)
Post-ICI	82	52.83 (35.68, 68.16)	0.44 (0.00, 1.25)	0.99 (0.97，1.00)	7.07 (6.16, 8.37)
*P*-value		<0.001	0.012	0.636	<0.001

## Penile curvature and Etiology of ED

### Relationship between penile curvature and ED by Etiology

Among the 82 ED patients, 55 (67.07%) were non-vascular, and 27(32.93%) were vascular. In the non-vascular group, 24 had concomitant penile curvature and 31 did not. In the vascular group, 10 had curvature while 17 did not. Statistical analysis revealed no significant association between the presence of penile curvature and the etiology of ED (*P* > .05) (Table 2).

**Table 2:** Penile Curvature and Etiology of ED.

Etiology of ED	With penile curvature	Without penile curvature
non-vascular	10	17
vascular	24	31
Pearson Chi-Square X^2^	0.33	
*P-*value	.57	

### Comparison of hemodynamic and elastic parameters between non-vascular and vascular etiologies in ED patients with comorbid penile curvature

Among 34 patients with ED concomitant with penile curvature, there were 24 cases of non-vascular etiology and 10 cases of vascular etiology.

During ICI-induced erection, there were significant differences (*P* < .05) in hemodynamic parameters while no significant differences were observed in elastic parameters between the vascular group (*n* = 10, 29.41%) and the non-vascular group (*n* = 24, 70.59%) (*P* > .05) (Table 3).

**Table 3:** Comparison of the post-ICI hemodynamic parameters and elasticity values between non-vascular group and vascular group in patients with ED concomitant with penile curvature.

Etiology	No.	PSV (cm/s)	EDV (cm/s)	RI	YM (kpa)
non-vascular	24	59.80 (50.56, 75.24)	0.37 (0.00，0.74)	0.99 (0.98，1.00)	6.92(6.29, 8.27)
vascular	10	27.67 (22.93, 43.41)	1.39 (0.60，2.20)	0.95 (0.94，0.98)	7.59(5.93, 10.48)
*P-*value		<.01	<.01	<.01	.68

## Comparisons of non-curvature group and curvature group

In this study, patients were divided into two distinct groups by post-ICI curvature status: comprising 34 curvature cases (41.46%) and 48 non-curvature cases (58.54%).

Hemodynamic and corporal stiffness profiling revealed non-significant elevations in pre-/post-ICI PSV, EDV, pre-ICI YM metrics, and slight reductions in post-ICI YM of curvature group compared to non-curvature group (*P* > .05). Comprehensive parametric comparisons are detailed in Table 4**.**

**Table 4:** Comparison of the blood flow parameters and elasticity values between non-curvature group and curvature group.

		Pre-ICI				Post-ICI			
	No.	PSV (cm/s)	EDV (cm/s)	RI	YM (kpa)	PSV (cm/s)	EDV (cm/s)	RI	YM (kpa)
curvature group	34	12.00 (8.98, 15.46)	0.00 (0.00，0.79)	1.00 (0.93，1.00)	24.20 (17.44, 30.40)	54.09 (38.29, 74.35)	0.57 (0.08, 1.36)	0.99 (0.97, 1.00)	6.92 (6.14, 8.44)
non-curvature group	48	10.78 (8.06, 13.63)	0.00 (0.00，0.64)	1.00 (0.94，1.00)	22.48 (15.60, 33.70)	49.57 (35.34, 64.42)	0.41 (0.00, 1.14)	0.99 (0.97, 1.00)	7.11 (6.16, 8.26)
*P-*value		.13	.78	.74	.83	.43	.32	.49	.91

## Comparisons among different penile curvature angles groups and different penile curvature directions groups

### Angulation-dependent hemodynamic and biomechanical variations

#### Group analysis by penile curvature and severity

Post-ICI evaluation stratified 82 pharmacologically induced erections into curvature severity cohorts: clinical insignificant curvature group (*n* = 50, 60.98%), mild curvature group (*n* = 12, 14.63%), and moderate–severe curvature group (*n* = 20, 24.39%). Pre-ICI hemodynamic analysis demonstrated significant intergroup variations in EDV and RI (*P* < .05), while PSV and YM showed non-significant differentials. (Notably, all post-ICI parameters demonstrated non-significant intergroup variations (*P* > .05). Comprehensive angulation-dependent parameter distributions are tabulated in Table 5.

**Table 5:** Comparison of the blood flow parameters and elasticity values among different penile curvature angles groups.

		Pre-ICI				Post-ICI			
Curvature group	No.	PSV (cm/s)	EDV (cm/s)	RI	YM (kpa)	PSV (cm/s)	EDV (cm/s)	RI	YM (kpa)
insignificant	50	10.78 (8.09, 13.58)	0.00 (0.00，0.66)	1.00 (0.94，1.00)	22.48 (15.36, 33.90)	49.57 (35.68, 64.41)	0.35 (0.00, 0.94)	0.99 (0.97, 1.00)	7.11 (6.16, 8.34)
mild	12	13.63 (10.19, 15.18)	0.00 (0.00，0.00)	1.00 (1.00，1.00)	23.30 (18.11, 30.11)	54.42 (42.31, 75.39)	0.54 (0.16, 1.28)	0.99 (0.97, 0.99)	6.92 (6.11, 7.83)
moderate or severe	20	12.00 (8.08, 17.40)	0.30 (0.00, 1.34)	0.98 (0.92, 1.00)	24.75 (18.03, 35.33)	56.31 (27.08, 74.24)	0.63 (0.03, 1.40)	0.98 (0.96, 1.00)	7.08 (6.12, 8.75)
*P-*value		.25	.01	.02	.89	.60	.41	.55	.74

#### Comparison by curvature degree in subjects with significant penile curvature

While insignificant penile curvature during erection can be found in some normal males and does not warrant clinical assessment, significant curvature requires evaluation. Intervention is typically considered when the curvature exceeds 30° due to potential sexual dysfunction. Therefore, we conducted a secondary analysis specifically on individuals with significant curvature.

**Figure 5:**
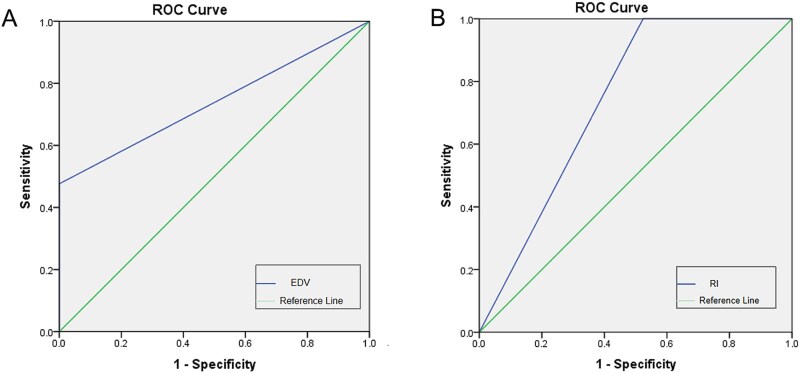
Receiver operating characteristic curve analysis of mild and moderate–severe curvate groups. (A) Receiver operating characteristic curve of EDV between mild and moderate–severe curvate groups. The area under the receiver operating characteristic curve is 0.75 (95% CI: 0.58-0.92). (B) Receiver operating characteristic curve of RI between mild and moderate–severe curvate groups. The area under the receiver operating characteristic curve is 0.75(95% CI: 0.58-0.92).

Pre-ICI hemodynamic analysis revealed significant differences in EDV and RI between the mild curvature group (*n* = 12, 14.63%) and the moderate–severe curvature group (*n* = 20, 24.39%) (EDV: area under the receiver operating characteristic curve AUC = 0.75; 95% CI: 0.58-0.92, *P* = .02; RI: AUC = 0.75; 95% CI: 0.58-0.92, *P* = .02). No significant differences were observed in PSV and YM. Furthermore, no parameters showed significant differences between the two groups post-ICI (*P* > .05) (Table 6 and Figure 5).

**Table 6:** Comparison of the blood flow parameters and elasticity values between mild curvature group and moderate–severe curvature group.

		Pre-ICI				Post-ICI			
Curvature group	No.	PSV (cm/s)	EDV (cm/s)	RI	YM (kpa)	PSV (cm/s)	EDV (cm/s)	RI	YM (kpa)
mild	12	13.63 (10.19, 15.18)	0.00 (0.00，0.00)	1.00 (1.00，1.00)	23.30 (18.11, 30.11)	54.42 (42.31, 75.39)	0.54 (0.16, 1.28)	0.99 (0.97, 0.99)	6.91 (6.11, 7.83)
moderate or severe	20	12.00 (8.08, 17.40)	0.30 (0.00, 1.34)	0.98 (0.92, 1.00)	24.75 (18.03, 35.33)	56.31 (27.08, 74.24)	0.63 (0.03, 1.40)	0.98 (0.96, 1.00)	7.08 (6.12, 8.75)
*p* value		0.89	<0.01	<0.01	0.53	1.00	0.74	0.81	0.51

The optimal EDV cutoff for distinguishing mild from moderate–severe curvature was 0.30, with a Youden’s index of 0.50, sensitivity of 50%, and specificity of 100%. This suggests that an EDV >0.30 is associated with a higher likelihood of moderate–severe curvature post-ICI, potentially requiring clinical intervention.

For RI, the identified cutoff was 0.98 (Youden’s index = 0.50, sensitivity = 100%, specificity = 50%). An RI <0.98 indicates a lower probability of mild curvature post-ICI.

### Direction-dependent hemodynamic and biomechanical variations

Systematic documentation of curvature direction vectors in 34 patients post-ICI revealed six directional subgroups: left lateral group (*n* = 10), left dorsal group (*n* = 8), right lateral group (*n* = 6), right dorsal group (*n* = 2), pure dorsal group (*n* = 4), and ventral group (*n* = 4). Biomechanical assessment demonstrated significant directional variations in YM among different penile curvature directions groups (*P* < .05)，with ventral curvatures exhibiting higher YM values, while dorsal curvatures presenting lower stiffness. Detailed directional hemodynamic and biomechanical characteristics are quantified in Table 7.

**Table 7:** Comparison of the blood flow parameters and elasticity values among different penile curvature directions groups.

		Pre-ICI				Post-ICI			
Curvature group	No.	PSV (cm/s)	EDV (cm/s)	RI	YM (kpa)	PSV (cm/s)	EDV (cm/s)	RI	YM (kpa)
left curve	10	13.85 (11.61, 16.61)	0.00 (0.00，1.38)	1.00 (0.93，1.00)	27.38 (18.63, 30.40)	48.80 (29.10, 67.16)	0.50 (0.00, 1.20)	0.99 (0.95, 1.00)	7.75 (6.37, 8.58)
left dorsally curved	8	11.65 (7.59, 14.37)	0.00 (0.00，0.53)	1.00 (0.97，1.00)	27.05 (22.58, 36.33)	66.13 (47.08, 75.56)	0.92 (0.27, 1.85)	0.98 (0.96, 0.99)	6.13 (5.97, 6.98)
right curve	6	11.08 (7.75, 15.71)	0.00 (0.00, 0.00)	1.00 (1.00, 1.00)	19.13 (14.98, 25.28)	61.93 (50.98, 79.21)	0.47 (0.10, 0.62)	0.99 (0.99, 1.00)	7.25 (6.73, 7.88)
right dorsally curve	2	10.60 (5.90, 10.60)	0.38 (0.00, 0.38)	0.93 (0.86, 0.93)	21.75 (21.15, 21.75)	50.34 (26.40, 50.34)	4.05 (1.42, 4.05)	0.93 (0.91, 0.93)	7.22 (5.98, 7.22)
dorsal curve	4	9.90 (7.02, 16.30)	0.30 (0.00, 1.31)	0.96 (0.92, 1.00)	14.38 (8.19, 18.98)	34.43 (23.83, 91.04)	0.68 (0.00, 1.36)	0.99 (0.97, 1.00)	5.52 (3.74, 11.04)
ventral curve	4	11.65 (10.33, 16.35)	1.13 (0.23, 1.88)	0.90 (0.83, 0.99)	32.93 (29.21, 41.40)	52.86 (30.22, 70.08)	0.40 (0.16, 0.63)	0.99 (0.98, 0.99)	9.08 (7.19, 21.57)
*P-*value		.71	.19	.13	.03	.57	.30	.28	.18

*Note*: There were 2 patients with right dorsally curve, therefore the values were showed in the table，instead of inter-quartile range.

## Discussion

The physiological process of penile erection involves a complex interaction among neural, endocrine, and vascular systems. When sexual stimulation is accepted by the male body, neural signals are rapidly transmitted to the central nervous system, and subsequently triggers the peri-cavernous nerves to release the neurotransmitters, inducing relaxation of smooth muscle cells. The resultant hemodynamic changes facilitate substantial blood influx into the cavernosum, accompanied by rapid expansion of sinusoid lacunar spaces, culminating in penile erection. The engorgement of sinusoid lacunae mechanically compresses venous drainage pathways, and thereby maintaining erectile rigidity.^19^ Erectile dysfunction may occur when any factor in this cascade becomes compromised, including neural conduction, neurotransmitter release, or vascular smooth muscle function. During the erection process, asymmetrical filling of cavernous sinusoids or structural anomalies such as tunica albuginea fibrosis may lead to pathological penile curvature during erection.

In this study, the ICI of vasodilator drugs were used in our subjects, which has been proven to be useful for evaluation and treatment of patients with ED.^20^^,^^21^ Firstly, ED patients frequently exhibit suboptimal erectile responses to isolated sexual stimulation, and there may be insufficient clinical assessment of the severity of ED patients, which may affect subsequent therapeutic decision-making. Concurrently, ICI administration enables systematic evaluation of individual, response of ED patients to vasodilator drugs, thereby facilitating the development of personalized treatment protocols. Notably, in patients with comorbid ED and penile curvature, suboptimal erectile attainment may compromise the accuracy of curvature quantification. Substantial evidence as documented across multiple comparative studies confirms significant discrepancies between curvature degree metrics derived from ICI-induced versus naturally achieved erections.^22^ Therefore, precise evaluations of both ED severity and penile curvature angle are paramount for optimizing treatment strategies. Post-ICI assessment becomes indispensable, especially in patients with severe ED or significant penile curvature.

Multimodal ultrasound imaging was employed to assess structural integrity, hemodynamic parameters, and elastic properties of the penile corpora cavernosa post-ICI, which provide valuable diagnostic and therapeutic insights.

High-frequency ultrasound examination can clearly show the layered structure of the penis, with a high detection sensitivity for penile soft tissue lesions, including benign and malignant epidermal neoplasms, traumatic defects, and corpus cavernosum discontinuities. In our cohort of 82 patients, Peyronie’s disease was identified in 8 patients (9.76%), with 6 of these patients experienced penile curvature. Anatomical correlation revealed lesions localization ipsilateral to penile curvature orientation in 5 patients supporting Peyronie’s disease induced fibrosis mechanical tension as a primary etiological factors. One exceptional case demonstrated contralateral cavernosal lesion localization relative to the curvature degree, presenting with mild deformity of 20°, this finding suggested potential multifactorial pathogenesis involving both fibrotic changes of corpus cavernosum and additional biomechanical determinants. When evaluating patients with a penile curvature angles >30°, we should carefully observe the layered structure of the penis through high-resolution ultrasound modalities to assess underlying structural etiologies.

Color Doppler ultrasound and shear wave elastography serve as valuable diagnostic modalities in penile evaluation. Our observations revealed that following ICI-induced penile erection, the PSV of penile cavernous body blood flow demonstrated significantly increased, while EDV showed slight elevation, findings consistent with established physiological mechanisms of penile erection characterized by increased penile perfusion. SWE measurements exhibited distinct patterns, with penile erecilet state values being lower than in the flaccid state. This discrepancy could be explained by structural modifications during tumescence: the expansion of the corpus cavernosum sinusoid lacunae altered the proportion of smooth muscle and collagen fibers per unit area. Notably, YM values mainly reflected the mechanical properties of blood-filled sinusoid lacunae, which demonstrated lower elasticity values compared to dense stromal tissues, which was consistent with previous study results of Ailin Cui.^23^^,^^24^

Regarding the etiology of ED, we classified it into non-vascular and vascular causes. In our study, we found that the presence of penile curvature showed no direct correlation with the etiology of ED. However, among patients with penile curvature, significant differences in hemodynamic parameters were observed between the non-vascular and vascular groups. This finding is consistent with numerous previous studies and underscores the utility of these parameters in differentiating between non-vascular and vascular ED.^25^ In contrast, elasticity parameters demonstrated no significant differences between the two groups.

In this study, we compared the parameters of patients with varying degrees and directions of penile curvature. Comparative analysis between the curved and non-curved penile groups revealed no statistically significant differences in post-ICI hemodynamic parameters and elastic parameters. We hypothesized that the alterations in penile structure and hemodynamics might only become clinically detectable beyond a specific curvature threshold. This premise prompted us to perform a stratified analysis based on the angle of deviation and vector direction, followed by subsequent subgroup comparisons. Notably, significant differences in pre-erectile EDV and RI values were observed across distinct penile curvature angles, while pre-erectile YM values exhibited divergence among patients with different penile curvature directions. These observations indicated that the potential utility of pre-erectile hemodynamic and elastographic parameters and elastic parameters in predicting post-erectile curvature patterns, including angular magnitude and directional characteristics. In patients with ED and significant penile curvature, even without ICI assessment, we can employ multimodal ultrasound to measure penile arterial EDV and RI values for a predictive evaluation. When the EDV is greater than 0.30 m/s and the RI is less than 0.98, there is a high suspicion that post-erection moderate–severe curvature will occur, which may compromise sexual satisfaction. This finding warrants further assessment to determine the need for clinical intervention. It was important in clinical assessment, especially for patients with ED, who frequently presented insufficient spontaneous erection and needed to undergo other examinations or even invasive procedures to fully evaluate their post-erectile status.

This study had several limitations. First, the lack of histopathological validation prevented definitive confirmation of our findings. While SWE was employed to evaluated penile curvature characteristics in ED patients, based on many previous studies suggesting that elastographic discrepancies might correlate with corporal structural characteristics, compositional variations, or proportional differences caused by penile curvature, while direct histopathological validations remained lacking. Second, the subgroup analysis based on the direction of penile curvature was limited by a small sample size, which may constrain the robustness of our findings. Future studies should enroll a larger cohort to enable a more comprehensive analysis. Third, although performed by an experienced andrologist, the use of a manual goniometer is inherently susceptible to inter-observer variability and measurement error, despite our precautions of having two practitioners present and employing a standardized goniometer for direct measurement. Finally, the single-center design with a small sample size necessitated cautious interpretation of findings. Subsequent investigations should be pursued to expand recruitment and potentially multi-center collaborative investigations should be organized to enhance generalizability.

## Conclusion

In conclusion, during the clinical evaluation of patients presenting with ED accompanied by penile curvature, comprehensive management should address both anatomical deformity correction and functional restoration. Post-ICI assessment enables more comprehensive evaluation of erectile capacity and curvature characteristics, while multi-modal ultrasounography can provide valuable insights into potential etiological factors. SWE also emerges as a particularly valuable diagnostic tool for quantitatively evaluating curvature severity and erectile function in affected patients.
